# Guideline- Versus Non-Guideline-Based Neoadjuvant Management of Clinical T4 Rectal Cancer

**DOI:** 10.3390/curroncol30100676

**Published:** 2023-10-21

**Authors:** Xi Chen, Xinyu Xie, Xiaodong Wang, Mingtian Wei, Zhigui Li, Li Li

**Affiliations:** 1 Division of Gastrointestinal Surgery, Department of General Surgery, West China Hospital of Sichuan University, Chengdu 610041, China; cxchenxi@stu.scu.edu.cn (X.C.); 2019151650062@stu.scu.edu.cn (X.X.); 2 West China School of Medicine, West China Hospital of Sichuan University, Chengdu 610041, China; 3 Colorectal Cancer Center, West China Hospital of Sichuan University, Chengdu 610041, China; mtweiyy15@wchscu.cn (M.W.); lizhigui07@wchscu.cn (Z.L.); drlili116@126.com (L.L.)

**Keywords:** guideline, rectal cancer, neoadjuvant therapy

## Abstract

(1) Background: Practice guidelines recommend neoadjuvant treatment for clinical T4 rectal cancer. The primary objective of this retrospective study was to assess whether compliance with guidelines correlates with patient outcomes. Secondarily, we evaluated predictors of adherence to guidelines and mortality. (2) Methods: A total of 397 qualified rectal cancer (RC) patients from 2017 to 2020 at West China Hospital of Sichuan University were included. Patients were divided into two groups depending on adherence to neoadjuvant treatment guidelines. The main endpoints were overall survival (OS) and disease special survival (DSS). We analyzed factors associated with guideline adherence and mortality. (3) Results: Compliance with guidelines was only 39.55%. Patients’ neoadjuvant therapy treated not according to the guidelines for clinical T4 RC was not associated with an overall survival (95.7% vs. 88.9%) and disease special survival (96.3% vs. 91.1%) benefit. Patients were more likely to get recommended therapy with positive patient compliance. Staging Ⅲ, medium/high differentiation and objective compliance were associated with increased risk of mortality. (4) Conclusions: Guideline adherence for clinical T4 RC in our system is low. Compliance with the relevant guidelines for neoadjuvant therapy seems not to lead to better overall survival for patients with clinical T4 RC.

## 1. Introduction

Rectal cancer (RC) is one of the most common cancers worldwide. According to global estimates of new cases and deaths, RC ranks as the third most common cancer in terms of incidence, and second in terms of mortality [1]. In 2020, colorectal cancer (CRC) ranked third in the most common types of cancer-related new cases in China in and fifth in the distribution of deaths [2].

National treatment guidelines for CRC were first published in China in 2015 and last updated in 2020 [3]. The guidelines are based on evidence-based medicine, including standardized clinical diagnosis, treatment methods following basic principles, and reasonable postoperative follow-up and monitoring. They are important reference guidelines for the clinical diagnosis and treatment of CRC. The intention of guidelines is to guide, rather than enforce, and so, the extent of their uptake varies [4]. Because of differences in regional development, doctors’ mastery of the guidelines, patient compliance and other reasons, it is difficult to follow detailed guidelines in clinical practice [5].

Both the National Cancer Comprehensive Network (NCCN) of the United States and the Oncology Branch of the Chinese Medical Association recommend neoadjuvant therapy as one of the treatments for advanced CRC [6]. The indications and schemes of neoadjuvant therapy for RC were also given. Nearly 50% of all RC patients had an assessable TN category and were classified as locally advanced rectal cancer (LARC) [7]. However, the formulation of guidelines and norms for the diagnosis and treatment of CRC in China is lagging behind, and clinicians have different mastery and compliance levels. A major question is whether deviation from current guidelines for neoadjuvant therapy is justified in patients with clinical T4 RC. However, there are few studies on the Chinese rectal cancer patient population. Therefore, the author’s team tried to use the whole life cycle data of RC from the CRC database of Gastrointestinal Surgery Center of West China Hospital of Sichuan University to evaluate the efficacy of preoperative, intraoperative and postoperative treatment of patients with resectable clinical T4 RC.

The objective of this study was to assess the impact of adherence to the Chinese protocol for the diagnosis and treatment of CRC (2020 edition) on the overall survival of patients with clinical T4 rectal tumors who underwent curative surgical excision and to compare the prognoses of patients following and not following guidelines for neoadjuvant treatment.

## 2. Materials and Methods

### 2.1. Study Design

This trial received Research Ethics Board approval at the clinical Trials and Biomedical Ethics committee of West China Hospital (2021-1690) and was registered on the Chinese Clinical Trial Registry (ChiCTR2200060746, registered on). Patient informed consent was waived because this study was retrospective and the data used were not individually identifiable.

Disease characteristics were assessed by clinical tumor-node-metastasis (TNM) classification (AJCC 8th). Clinical TNM staging was performed for preoperative assessment, and pathological TNM staging was used for patients after surgery. Variables used to describe the treatment process included neoadjuvant therapy, surgery, adjuvant therapy, and follow-up. Patients were contacted by telephone or social media by 31 December 2021 (end of study) and their status was determined: alive or not. Survival time (months) was defined as the duration from the date of operation to the end of follow-up or from the date of operation to the date of death for the analysis of overall survival and disease-specific survival. Treatment they received was extracted from medical records and compared to the Chinese protocol for the diagnosis and treatment of CRC (2020 edition). The consistency between the recommendations of the guidelines for neoadjuvant therapy, surgical methods and adjuvant therapy for CRC and the actual rate of provision of these treatments was analyzed. Patients were divided into two groups: the guideline-treated group and the non-guideline-treated group according to whether they received standard therapy or not. A summary of treatment recommendations and discrepancies from the non-guideline-treated group is shown in Table 1.

Patient compliance was grouped into “passive, objective and positive”, which meant patients’ attitudes towards the neoadjuvant treatment options proposed by their doctors were “passive acceptance”, “refusal” and “active cooperation”. Based on criteria in the guidelines, 0–49% glandular duct formation was defined as low differentiation, 50–95% duct formation was defined as medium differentiation and >95% duct formation was defined as high differentiation.

### 2.2. Eligibility Criteria

All patients met the following criteria: (1) 18 years of age or older; (2) well-preserved main organ functions; (3) a pathologically confirmed diagnosis of RC; (4) underwent total mesenteric resection (TME); and (5) clinical T4 primary rectal adenocarcinoma.

### 2.3. Primary/Secondary Endpoint and Statistical Analysis

#### 2.3.1. Primary/Secondary Endpoint

The primary endpoint was overall survival (OS), which was defined as survival from registration to death by any cause. The patients were censored if no events occurred at the final data cutoff. The secondary endpoint was disease-specific survival (DSS), which was defined as the percentage of people in a study or treatment group who had not died from a specific disease in a defined period of time.

#### 2.3.2. Statistical Analysis

Differences in baseline characteristics between those treated according to guidelines and those treated otherwise were analyzed using the Chi-square test and *t*-test.

To evaluate the effect of each parameter on survival, survival rate estimation was performed using the Kaplan–Meier method, binary logistic regression analysis and Cox proportional hazards model. The results were assumed to be statistically significant if the *p*-value < 0.05. SPSS Statistics 26.0 (IBM, Armonk, NY, USA) and R studio were used for statistical analysis.

## 3. Results

### 3.1. Patient Characteristics

A total of 397 patients receiving surgery for T4 rectal cancer between 2017 and 2021 were identified from the CRC database of the Division of Gastrointestinal Surgery, Department of General Surgery, West China Hospital of Sichuan University, and they were selected and distributed into two groups according to whether they received standard therapy or not; 157 patients (40%) were treated according to the guidelines, while 240 (60%) were not. The median age of the total population was 61 years (range 24–95 years), and 60% of patients were male. According to TNM staging, most patients were diagnosed with stage III disease (70%), followed by stage II (30%). In T4, there were 235 cases of T4a (59%) and 162 cases of T4b (41%). Based on BMI, 6% were classified as lean, 52% were classified as normal weight, 32% as overweight and 10% as obese. The median duration of follow-up time was 29 months in this group, ranging from 1 to 58 months. Positive compliance was significantly more often documented in patients treated according to the guidelines (*p* < 0.0001, Table 2).

### 3.2. Survival

**Figure 1:**
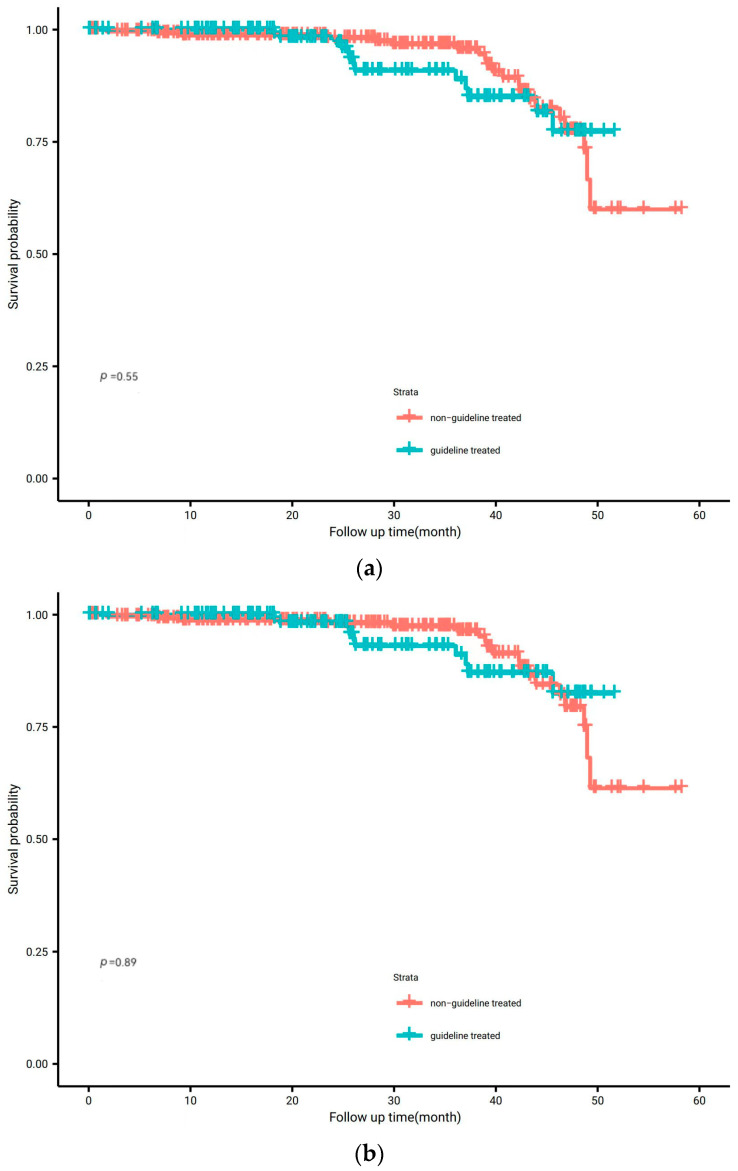
(**a**) Overall survival according to group (Log-Rank test). Kaplan–Meier overall survival curves comparing the guideline-treated group and the non-guideline-treated group; (**b**) disease-specific survival according to group (Log-Rank test). Kaplan–Meier overall survival curves comparing the guideline-treated group and the non-guideline-treated group.

Patients who received non-guideline care did not have reduced OS compared with patients who received neoadjuvant therapy according to the guidelines (Figure 1a), with a 3-year OS rate of 95.7% (95% confidence interval (CI):92.4–99.0%) vs. 88.9% (95% CI: 82.1–96.3%) (*p* = 0.55). A similar result was seen in the DSS analysis (Figure 1b). No significant survival benefit was demonstrated when comparing those who did not receive recommended guideline treatment in the Kaplan–Meier survival curves, with a 3-year DSS rate of 96.3% (95% CI: 93.3–99.4%) vs. 91.1% (95% CI: 84.7–97.9%) for other guideline-treated cases (*p* = 0.89).

### 3.3. Predictors of Adherence to Guidelines

A binary logistic regression analysis was performed to predict compliant care (Table 3). We found that patients with positive compliance were more likely to receive compliant care compared to patients with passive compliance (OR = 4.666; 95% CI: 2.726–7.987; *p* < 0.0001).

### 3.4. Predictors of Mortality

To determine the contributory role of guideline compliance to disease-specific survival in the context of other demographic and pathologic factors, a Cox regression multivariate analysis was performed for both cohorts (Table 4). The clinical TNM staging, differentiation and patient compliance were independent predictors of survival. Patient refusal to cooperate was associated with an increased risk of mortality (HR = 79.703; 95% CI: 13.294–477.833; *p* < 0.0001). Similarly, patients who were at clinical TNM stage III had a significantly increased odds of mortality (HR = 12.541; 95% CI: 2.377–66.167; *p* = 0.003). Conversely, higher differentiation was associated with an decreased risk of mortality, and patients with medium and high differentiation had better survival compared to those of low differentiation (HR = 0.06; 95% CI: 0.05–0.796; *p* = 0.033) (HR = 0.008; 95% CI: 0.000–0.301; *p* = 0.009). No period effect of guideline adherence on disease-specific survival was observed in multivariate analysis (HR = 0.507; 95% CI: 0.012–7.095; *p* = 0.351).

## 4. Discussion

The aim of our study was to evaluate the effect of compliance with guidelines on the prognosis of patients with clinical T4 RC in West China Hospital of Sichuan University. The results of the study demonstrated that nearly two-thirds of patients did not receive guideline-based neoadjuvant therapy, and the compliance with guidelines of preoperative neoadjuvant therapy had no significant impact on patient survival. This study explored multiple factors that contributed to nonadherence to the guideline-based delivery of neoadjuvant for patients with clinical T4 RC.

The NCCN and Oncology Branch of the Chinese Medical Association both recommend neoadjuvant therapy for advanced RC. Studies have also shown that hospitals with a high utilization rate of neoadjuvant therapy are associated with better survival [8,9,10]. However, in this study, only 39.55% (*n* = 140) of the patients received standard neoadjuvant therapy, which implies that current guidelines cannot be carried out easily for patients with clinical T4 RC. The reasons for the small proportion of RC cases receiving guideline-recommended neoadjuvant therapy may be multifactorial, reflecting the greater case complexity associated with the treatment of these cancers. Several similar studies have shown poor compliance with guidelines on the use of neoadjuvant therapy in practice, with only 50% of patients with stage III RC receiving neoadjuvant therapy before definitive surgery in a study in Sri Lanka in patients with absolute indications for neoadjuvant therapy for RC [11]. These findings are not unique. Many Asian countries including the Philippines, Japan, and Korea have also reported low rates of standard therapy use for RC [12]. Guideline adherence to neoadjuvant therapy in the US and the Netherlands was also not good [13,14]. Perhaps our findings are the result of physicians’ knowledge and mastery of guidelines varying widely, or because patients’ preferences differ, or for other reasons.

Despite these well-publicized treatment guidelines and a substantial body of evidence supporting the prognostic benefits of neoadjuvant radiotherapy for clinical T4 rectal cancer [6], the problems that can be reflected in the real world are removed from the nature of these guidelines and may encompass the characteristics of physicians and patients as actors. This study found that 3-year survival rates of clinical T4 RC patients receiving standard neoadjuvant therapy did not differ significantly from those who received other treatments. In terms of the comparison of overall survival curves, there was also no significant difference in survival rates between the two groups in this study. However, previous studies have shown that receiving guideline-based neoadjuvant radiotherapy resulted in better survival benefits within 5 years. The analysis of data from two teaching hospitals in the southern part of the Netherlands between 2008 and 2015 showed that patients treated according to the guidelines had better survival 18 months after diagnosis (80 versus 56%) [10]. Eid, Y. conducted a trial in 2019 that found compliance with the relevant guidelines improved the quality of the multidisciplinary management of patients who underwent curative surgery for subperitoneal RC [9]. These results are in contrast to our findings. Although our study showed that adherence to guidelines did not show significant survival outcomes in patients with T4 RC, this finding is in concordance with the following two reports. In 2018, a retrospective review of the National Cancer Data Base was carried out to demonstrate that patients younger than 50 years diagnosed with RC represent a unique demographic group in which the survival advantage of receiving NCCN guideline-driven therapy does not materialize [13]. Another Dutch trial found that under certain treatment quality standards, there might be different valid treatment options with similar oncological outcomes which may not have negative impacts on the quality of care [15].

Clinical practice guidelines are not prescriptive, and reasons for departing from guideline recommendations are multifaceted. We found that active patient cooperation facilitates the more standardized use of neoadjuvant therapy. It is important to communicate effectively with the patient so that they fully understand the treatment. Factors affecting guideline uptake would also include patient education, health insurance status, household income, etc. In addition, there may be sociological factors such as the convenience of off-site access for patients, whether there is adequate understanding and support for neoadjuvant treatment options [16,17].

Our results in multivariate analyses of prognostic factors also suggest that adherence to guidelines was not significantly correlated with the prognosis of RC. This is in line with other findings. A prospective study demonstrated a trend towards improved overall survival with neoadjuvant radiotherapy, but this was not significant in a multivariate analysis. Another study used COX regression multivariate analysis but did not find significant survival benefit for neoadjuvant therapy in patients under the age of 50 [13].

Although adherence to actual neoadjuvant treatment guidelines had no impact on patient prognosis, patients’ refusal of physician-recommended neoadjuvant regimens was an independent risk factor for prognosis, suggesting that clinical staff can develop effective interventions based on the above-mentioned factors influencing patient adherence and provide health education to patients in order to improve patient adherence to neoadjuvant treatment and improve patient prognosis. We also found that high clinical TNM staging and differentiation were associated with reduced overall survival in multivariate analysis. A higher stage means a higher degree of tumor progression. Pathology shows that as the degree of differentiation goes from high to low, the heterogeneity of rectal adenocarcinoma cells becomes progressively more significant and more malignant. Both lead to a worse prognosis. This observation is consistent with findings by other investigators [18,19].

Our findings highlight the operational difficulties of guideline-based therapies in the real world. It seems that many of the actual clinical practices are not perfectly consistent with the existing guideline recommendation. On the one hand, the apparent differences in the application of preoperative radiotherapy for those with clinical T4 may be related to a number of factors: down-staging following preoperative therapy, TNM staging itself and inaccurate preoperative staging. There may even be issues of medical subjective and objective diagnostic efficacy, such as the accuracy of imaging CT/MRI assessment of staging, clinical staging judgments on anal finger examination in patients with rectal cancer, the debate over high risk factors for colorectal cancer and whether hospitals and physicians have a comprehensive strategy for neoadjuvant treatment selection. On the other hand, we should consider that departures from guideline recommendations may be appropriate. Although TNM stage is an important influencing factor in determining preoperative treatment, there are many other factors that can affect the actual treatment plan of the surgeon. For example, in the CRC database of the Gastrointestinal Surgery Center of West China Hospital of Sichuan University, it is hard to understand the factors that drive clinical decision making, such as family attitudes, risk factors and exact clinical circumstances. Keeping these factors in mind, in some incidences, the omission of radiotherapy or deviation from guidelines might be warranted. Furthermore, hospitals and doctors may have a comprehensive strategy for the neoadjuvant treatment selection process. And the revised Dutch colorectal cancer guideline recommends that preoperative short-course radiotherapy should be considered as treatment for intermediate-risk rectal cancer, but that the benefits and risks should be discussed with the patient [20]. Physicians may deliver evidence-based cancer care and rapidly adopt evolving data into their clinical practice. Specialists’ experience and patient preferences contribute to a more individualized treatment of colorectal cancer, which does not always follow the principles of traditional evidence-based medicine. This makes the development and assessment of guidelines more difficult. Patient populations are increasingly subdivided into different clinical entities, and there are lots of multidisciplinary treatment options, leading to complex clinical decision making [18]. Based on the guidelines, it is important to thoroughly discuss options with the patient and determine the treatment policy. Further research should aim to adapt clinical practice or guidelines and improve clinical outcomes of rectal carcinoma.

This study has several limitations. First, this is a retrospective study, which is the main weakness of this study. It could lead to misinterpretation of the data, and it is subject to unmeasured, unnoticed bias and confounding factors that persist even with statistical correction. A certain degree of missing data is unavoidable in population-based studies, but these data could have been important, such as certain factors affecting treatment selection and follow-up. Patients were followed up for a relatively short period of time, with only a 3-year survival rate used in the study. A longer follow-up period would have facilitated our analysis of the patients’ long-term survival. It is also important to acknowledge that data were collected over an interval (2017–2021), which may also have introduced a degree of bias given that treatment strategies have evolved over time. Additionally, the study lacks external validation since it was monocentric. It only captured treatments performed at Gastrointestinal Surgery Center of West China Hospital, and data from other centers were not included, which may have different experiences and outcomes, limiting the generalizability. Therefore, further multicenter studies should be conducted. Finally, several elements in the data were not investigated in depth, such as reasons for deviations from guideline treatment. This lack of qualitative data suggested the need for further research on this topic.

Notwithstanding the limitations of this study, there are several important strengths. Life cycle data from the West China Hospital CRC Database of Sichuan University provide the availability of many clinically relevant variables in a unique population of cT4RC patients with a low prevalence of missing values. Second, this is a single-center study with detailed data in this specific population without inter-hospital variations. In addition, we specifically selected patients with clinical T4 rectal adenocarcinoma. Most previous studies have described patients with colon and RCs, and these patients require different treatment approaches. We also focused on the impact of the neoadjuvant module of the guidelines on clinical practice and patient prognosis, which is more relevant.

In conclusion, variability in the delivery of neoadjuvant chemoradiation in RC exists despite established guidelines. This retrospective cohort study illustrated that patients with clinical T4 RC who take neoadjuvant treatment according to guidelines do not gain more survival benefit. Compliance was correlated with decreased mortality. Measures should be taken to improve compliance in the care of these patients. There are many other factors in clinical practice that deviate from guidelines that were not explored in this study, and the need for the increased use of neoadjuvant therapy was not explored in this study, thus suggesting an area for further research. Potentially closer guideline alignment could be achieved, with improved clinical outcomes. Additionally, more prospective studies in patients with RC are needed to fine-tune the current guidelines, taking into account survival time and quality of life, which would contribute to shared clinical decision making.

## 5. Conclusions

This retrospective study showed a poor rate of compliance with guidelines for CRC in one of China’s largest hospitals. Patient compliance was associated with decreased mortality. Future studies should aim to improve the compliance in the neoadjuvant care of these patients.

## Figures and Tables

**Table 1:** Summary of treatment recommendations and discrepancies from non-guideline-treated group.

Stage	TNM	Treatment	Guideline Recommendations	Non-Guideline Treatment
II~III	cT4,N0~2,M0	Neoadjuvant treatment	Long-range simultaneous chemoradiotherapy/short-range radiotherapy/sequential neoadjuvant chemotherapy	Only chemotherapy/only radiotherapy/no intervention

**Table 2:** Baseline characteristics.

	Total (*n* = 397)	Non-Guideline-Treated (*n* = 241)	Guideline-Treated (*n* = 137)	*p* Value ^a^
Age, *n* (%)				0.814
≤45 years	42	27(64)	15(36)	
45–65 years	199	121(61)	78(39)	
≥ 65 years	156	92(59)	64(41)	
BMI, *n* (%)				0.547
<18.5 kg/m^2^	24	14(58)	10(42)	
18.5~23.9 kg/m^2^	207	127(61)	80(39)	
24.0~27.9 kg/m^2^	127	72(57)	55(43)	
≥28.0 kg/m^2^	39	27(69)	12(31)	
Sex, *n* (%)				0.052
Female	159	106(67)	54(34)	
Male	238	134(56)	103(44)	
cTNM, *n* (%)				0.149
II	120	79(66)	41(34)	
III	277	161(58)	116(42)	
Localization, *n* (%)				0.672
Low rectum	230	136(59)	94(41)	
Mid rectum	129	82(64)	47(36)	
High rectum	38	22(58)	16(42)	
Differentiation, *n* (%)				0.091
Low	13	7(54)	6(46)	
Medium	334	197(59)	137(41)	
High	15	13(87)	2(13)	
Patient compliance, *n* (%)				<0.0001 *
Passive	139	111(80)	28(20)	
Objective	15	14(93)	1(7)	
Positive	243	115(47)	128(53)	

* Indicates significant *p*-values. ^a^ Patients treated according to guidelines versus patients not treated according to guidelines.

**Table 3:** Binary logistic regression model predicting guideline compliance (rectal cancer, Gastrointestinal Surgery Center of West China Hospital of Sichuan University 2017–2021).

	OR	95% Confidence Interval	*p*
cTNM			
III (vs. II)	1.003	0.595–1.690	0.992
Sex			
Male (vs. female)	1.399	0.865–2.265	0.171
Age			
Age 45–65 years (vs. ≤45 years)	1.424	0.646–3.135	0.381
Age ≥65 years (vs. ≤45 years)	1.618	0.725–3.607	0.240
BMI			
BMI 18.5~23.9 kg/m^2^ (vs. <18.5 kg/m^2^)	1.032	0.403–2.643	0.947
BMI 24.0~27.9 kg/m^2^ (vs. <18.5 kg/m^2^)	1.481	0.552–3.971	0.435
BMI ≥ 28.0 kg/m^2^ (vs. <18.5 kg/m^2^)	0.746	0.220–2.525	0.637
Localization			
Mid rectum (vs. Low rectum)	0.811	0.491–1.338	0.412
High rectum (vs. Low rectum)	0.934	0.418–2.087	0.867
Differentiation			
Medium (vs. Low)	0.885	0.268–2.927	0.842
High (vs. Low)	0.182	0.026–1.286	0.088
Patient compliance			
Objective (vs. Passive)	0.385	0.047–3.177	0.376
Positive (vs. Passive)	4.666	2.726–7.987	<0.0001 *

* Indicates significant *p*-values.

**Table 4:** Multivariable Cox proportional hazards analysis of disease-specific survival.

	HR	95% Confidence Interval	*p*
cTNM			
III (vs. II)	12.541	2.377–66.167	0.003 *
Sex			
Male (vs. female)	1.340	0.339–5.292	0.676
age			
Age 45–65 years (vs. ≤45 years)	22.582	0.754–676.546	0.072
Age ≥ 65 years (vs. ≤45 years)	44.962	1.289–1568.445	0.036
BMI			
BMI 18.5~23.9 kg/m^2^ (vs. <18.5 kg/m^2^)	0.588	0.089–3.899	0.582
BMI 24.0~27.9 kg/m^2^ (vs. <18.5 kg/m^2^)	1.911	0.261–13.98	0.524
BMI ≥ 28.0 kg/m^2^ (vs. <18.5 kg/m^2^)	0.738	0.059–9.282	0.814
Localization			
Mid rectum (vs. Low rectum)	0.520	0.124–2.185	0.372
High rectum (vs. Low rectum)	0.294	0.012–7.095	0.451
Differentiation			
Medium (vs. Low)	0.060	0.005–0.796	0.033 *
High (vs. Low)	0.008	0.000–0.301	0.009 *
Patient compliance			
Objective (vs. Passive)	79.703	13.294–477.833	<0.0001 *
Positive (vs. Passive)	0.106	0.010–1.121	0.062
Guideline treatment (vs. non)	0.507	0.122–2.113	0.351

* Indicates significant *p*-values.
